# PSMD4 is a novel therapeutic target in chemoresistant colorectal cancer activated by cytoplasmic localization of Nrf2

**DOI:** 10.18632/oncotarget.25254

**Published:** 2018-05-29

**Authors:** Ya-Min Cheng, Po-Lin Lin, De-Wei Wu, Lee Wang, Chi-Chou Huang, Huei Lee

**Affiliations:** ^1^ Department of Obstetrics and Gynecology, National Cheng Kung University Hospital, College of Medicine, National Cheng Kung University, Tainan, Taiwan; ^2^ Graduate Institute of Cancer Biology and Drug Discovery, Taipei Medical University, Taipei, Taiwan; ^3^ Department of Public Health, Chung Shan Medical University, Taichung, Taiwan; ^4^ Department of Surgery, Chung Shan Medical University, Taichung, Taiwan

**Keywords:** PSMD4, chemoresistance, Nrf2, colorectal cancer

## Abstract

Nuclear Nrf2 (nNrf2) binding to the antioxidant response element may promote chemoresistance in colorectal cancer. However, the shuttling of Nrf2 between cytoplasm and nucleus in colon cancer cells has revealed the possibility that cytoplasmic location of Nrf2 (cNrf2) may play a specific role in chemoresistance. Transfection of a nuclear location sequence (NLS)-wild-type or NLS-mutated Nrf2 expression vector into a stable shNrf2 HCT116 clone using the MTT assay to examine whether chemoresistance induced by cNrf2 may be greater than nNrf2. Different specific inhibitors and small hairpin (sh)RNAs of targeting genes were used to verify the mechanistic action of cNrf2 in chemoresistance and further confirmed by an animal model. The association of cNrf2 with chemotherapeutic response in patients with colorectal cancer was statistically analyzed. The MTT assay indicated that cNrf2 may play a more important role than nNrf2 in conferring 5-fluorouracil (5-FU) and oxaliplatin resistance in HCT116 cells. Mechanistically, cNrf2-induced PSMD4 expression was responsible for chemoresistance in the NLS-mutated Nrf2-tranfected shNrf2HCT116 clone via the NF-κB/AKT/β-catenin/ZEB1 cascades. The tumor burden induced by the NLS-mutated Nrf2-transfected shNrf2HCT116 clone was completely suppressed by treatment with 5-FU in combination with carfilzomib. A higher prevalence of unfavorable chemotherapeutic response in colorectal cancer patients with cNrf2, PSMD4-positive, p-p65-positive, and nuclear β-catenin tumors was observed when compared to their counterparts. cNrf2 may play a more important role than nNrf2 in the chemoresistance of colorectal cancer. Activation of the NF-κB/AKT/β-catenin/ZEB1 cascade by PSMD4 may be responsible for cNrf2-mediated chemoresistance.

**Condensed abstract:**

CNrf2 may play a more important role than nNrf2 in conferring 5-FU and oxaliplatin resistance. This observation in patients seemed to support the findings of the cell and animal models and suggested that PSMD4 may be responsible cNrf2-mediated chemoresistance via the NF-κB/AKT/β-catenin /ZEB1 cascades.

## INTRODUCTION

Chemotherapy based on 5-fluorouracil (5-FU) is the first-choice therapeutic approach for patients with colorectal cancer [1–4]. However, chemoresistance and tumor relapse are common occurrences in patients who undergo this treatment, and consequently result in patients with poor prognosis [5, 6]. Therefore, reliable biomarkers or viable strategies that improve chemotherapeutic efficacy and patient outcomes are urgently needed.

Nrf2 is overexpressed in colorectal tumors, where it assumes both nuclear (nNrf2) and cytoplasmic (cNrf2) localizations [7]. The nNrf2 binding to the antioxidant response element (ARE) upregulates the expression of antioxidant genes, such as heme oxygenase-1 (HO-1) [8–10]. This then protects cancer cells from DNA damage and apoptosis induced by reactive oxygen species (ROS), thereby enabling cancer cell survival and growth [8–10]. Accumulating evidence now shows that nNrf2 mediated by the Nrf2/ARE pathway may promote tumor progression and drug resistance in different types of human cancers [11–17].

The shuttling of Nrf2 between nuclear and cytoplasmic locations in cancer cells may play a specific role in tumor progression and drug resistance. This possibility is suggested by the observations that more than half of colorectal tumors (75/139, 54%) show cytoplasmic localization of Nrf2 and that patients with cNrf2 tumors have poorer prognosis than those with nNrf2 tumors [7]. In addition, cNrf2 is a stronger promoter than nNrf2 for colony formation, soft agar growth, and invasiveness of colon cancer cells because it increases PSMD4 expression. PSMD4-mediated p53 degradation increases CRM1 expression, which then reciprocally elevates cNrf2 expression by enhancing Nrf2 export from the nucleus [7].

We further demonstrated that upregulation of PSMD4 at the transcriptional level by cNrf-2-induced HIF-1α and nuclear β-catenin persistently promoted cells with a more aggressive phenotype via the NF-κB/AKT/β-catenin/ZEB1 cascade [7]. We therefore hypothesized that cNrf2-induced PSMD4 expression may promote more than just colorectal cancer tumor aggressiveness and may also confer chemoresistance, consequently resulting in patients with poor outcomes. We provide evidence from cell and animal models in support of this hypothesis and suggest that targeting PSMD4 by the new-generation proteasomal inhibitor carfilzomib may be more effective at overcoming 5-FU resistance and suppressing the tumor burden in nude mice induced by cNrf2-overexpressing colon cancer cells.

## RESULTS

### The contribution to 5-FU and oxaliplatin resistance is greater for cNrf2 than for nNrf2 and the resistance can be overcome by carfilzomib

**Figure 1:**
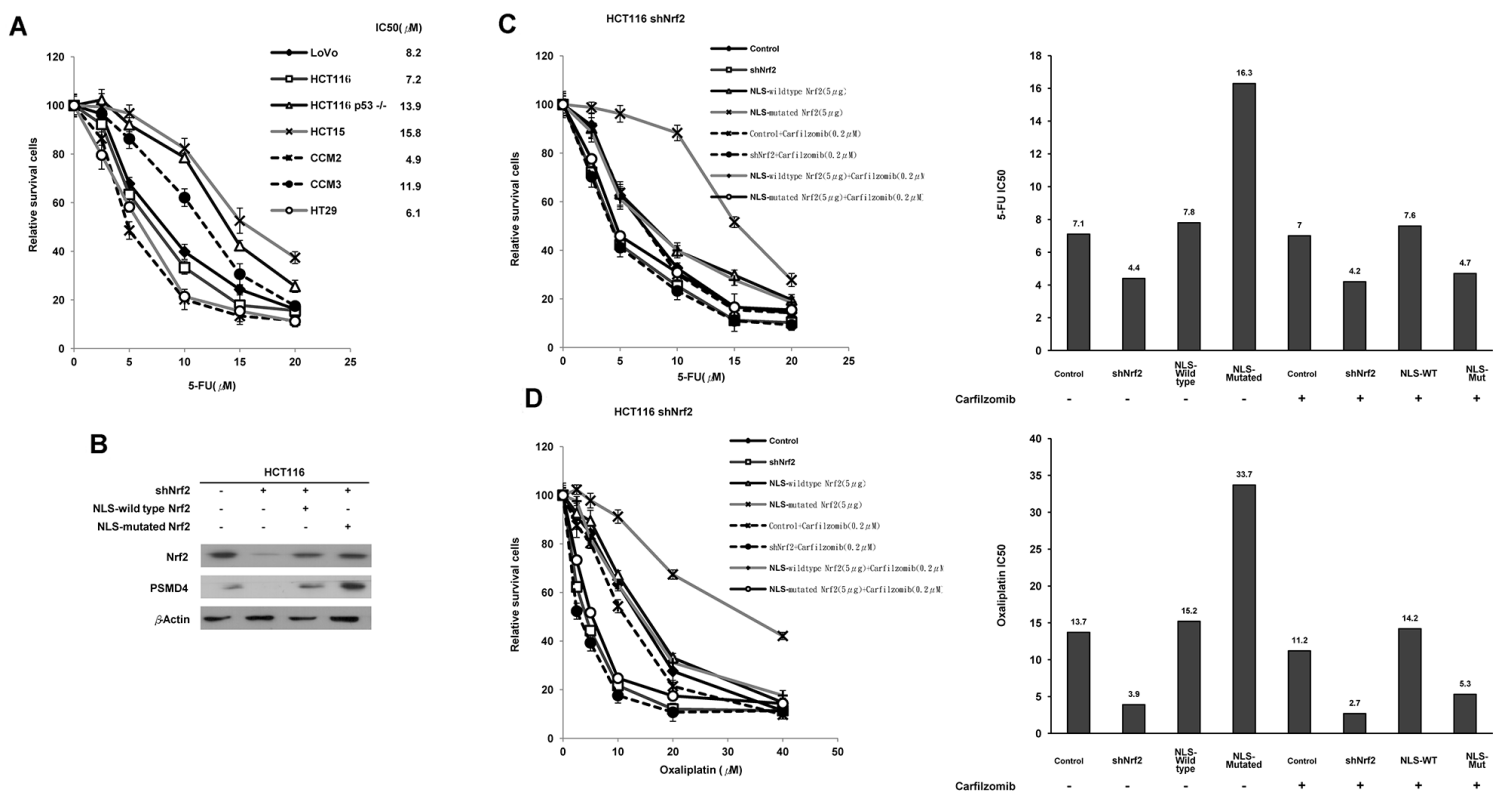
cNrf2 may contribute more than nNrf2 to 5-FU and oxaliplatin resistance and the resistance can be reversed by carfilzomib treatment **(A)** Colorectal cell lines were treated with six concentrations of 5-FU to calculate the IC50 value from the dose-response survival curve determined by the MTT assay. **(B)** NLS-WT Nrf2 or a NLS-mutated Nrf2 expression vector was transfected into a shNrf2-HCT116 stable clone. Western blotting analysis was performed to evaluate Nrf2 and PSMD4 expressions in different colon cancer cell lines. NLS-WT Nrf2 and NLS-mutated Nrf2 plasmids were transfected into the stable shNrf2-HCT116 clone. After 24 h, cells were treated with carfilzomib (0.2 μM) and then with six concentrations of **(C)** 5-FU and **(D)** oxaliplatin to calculate the IC50 value from the cell survival curves using the MTT assay.

Our previous report indicated that a higher Nrf2 expression was observed in LoVo, HCT15, HCT116, and HCT116 p53-/- cells than in CCM2, CCM3, and HT29 colon cancer cells [7]. We examined the possibility that the cells with higher Nrf2 expressing cells could have more contributive to 5-FU resistance than lower Nrf2 expressing cells. The inhibition concentration of 5-FU yielding 50% viability (IC50) of these cells was determined by the dose-response curve (Figure 1A). The highest IC50 value for 5-FU was observed in HCT15 cells (15.8 μM) followed by HCT116 p53-/- (13.9 μM), CCM3 (11.9 μM), LoVo (8.2 μM), HCT116 (7.2 μM), HT29 (6.1 μM), and CCM2 cells (4.9 μM). These results seemed to support the possibility that cNrf2 may contribute more than nNrf2 on 5-FU resistance in colon cancer cells.

We next used the Nrf2-knockdown HCT116 stable clone transfecting a NLS-wild-type (WT) Nrf2 or a NLS-mutated Nrf2 expression vector to verify whether cNrf2 could confer more 5-FU resistance than nNrf2 in colon cancer cells. Western blotting indicated that the expression of Nrf2 and PSMD4 concomitantly decreased; however, both molecules were increased by transfection of NLS-WT Nrf2 or NLS-mutated Nrf2. Interestingly, a 26S proteasome subunit PSMD4 showed a more significantly elevated expression in response to NLS-mutated Nrf2 than to NLS-WT Nrf2 in the shNrf2HCT116 clone (Figure 1B). The MTT assay indicated that the IC50 value for 5-FU was higher for the shNrf2HCT116 clone transfected with NLS-mutated Nrf2 (IC50 = 16.3 μM) than with NLS-WT Nrf2 (IC50 = 7.8 μM), but the IC50 value was significantly elevated when the shNrf2HCT116 clone was transfected with either expression vector and compared with shNrf2HCT116 cells transfected with an empty vector (VC, IC50 = 4.4 μM) (Figure 1C). The increase in the IC50 value for 5-FU by transfection of NLS-mutated Nrf2 into the shNrf2HCT116 clone was nearly reversed by a proteasomal inhibitor carfilzomib treatment (IC50 = 4.7 μM) (Figure 1C). The IC50 value for 5-FU in the parental HCT116 cells (Control), shNrf2HCT116 cells, and the NLS-WT-transfected shNrf2HCT116 clone was unchanged by carfilzomib treatment (7.1 vs. 7.0 μM for HCT116, 4.4 vs. 4.2 μM for shNrf2HCT116, 7.8 vs. 7.6 μM for NLS-WT-transfected shNrf2 HCT116; Figure 1C). Similar findings were also observed for the IC50 value for oxaliplatin in the shNrf2HCT116 clone subjected to the same treatments (Figure 1D). A similar decrease in the IC50 value for 5-FU and oxaliplatin by carfilzomib and MG-132, a standard proteasomal inhibitor, was observed in NLS-mutated Nrf2-transfected shNrf2HCT116 clone (Supplementary Figure 1). These results suggest that cNrf2 may contribute more than nNrf2 to 5-FU and oxaliplatin resistance and that this resistance can be reversed by carfilzomib treatment.

### Activation of NF-κB/AKT/β-catenin cascade by cNrf2-induced PSMD4 expression may be responsible for 5-FU resistance due to increased ZEB1 expression

**Figure 2:**
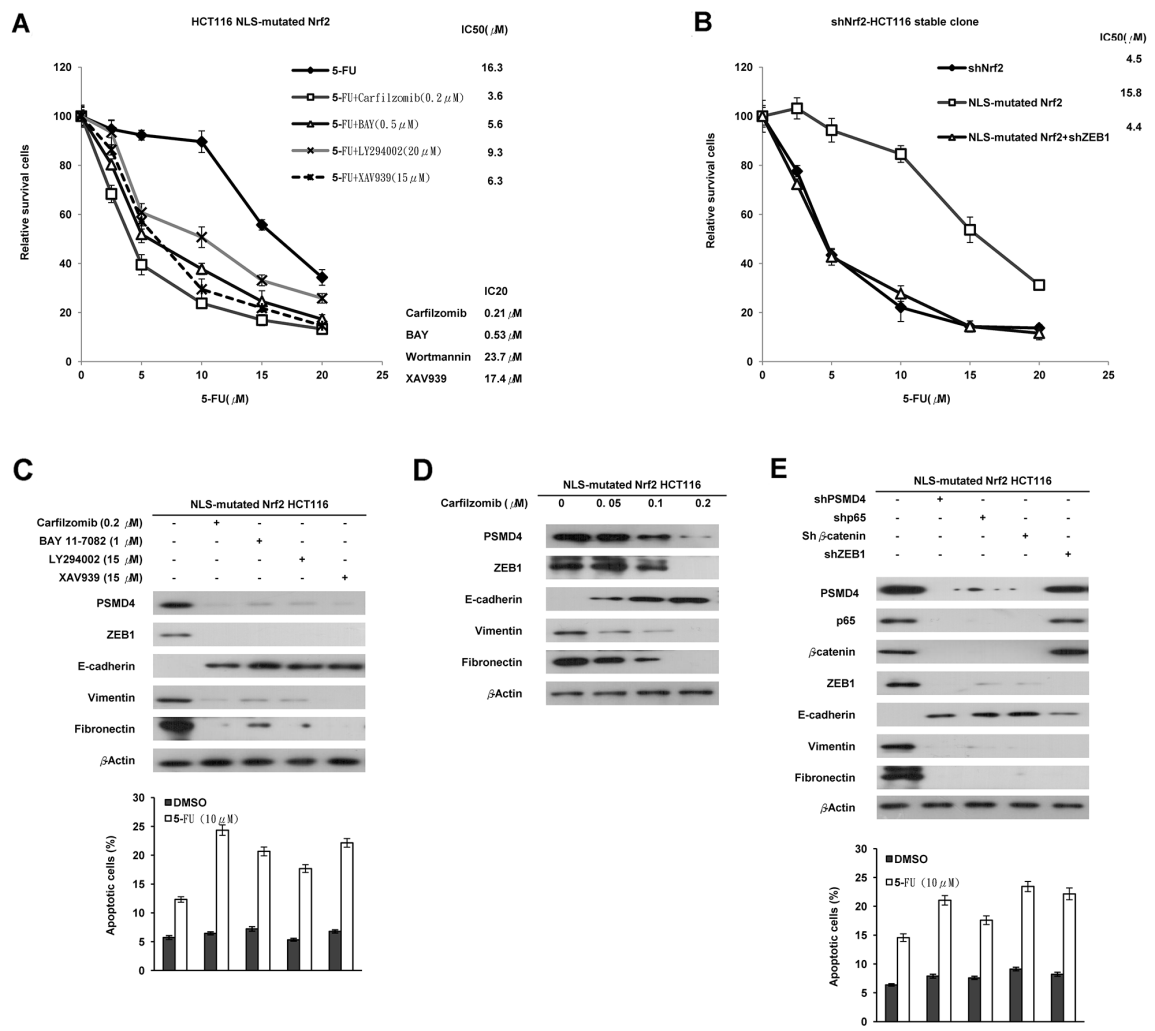
Activation of the NF-κB/AKT/β-catenin cascade by cNrf2-induced PSMD4 expression may be responsible for 5-FU resistance due to increased ZEB1 expression **(A)** The NLS-mutated Nrf2 plasmid was transfected into the shNrf2-HCT116 clone. After 24 h, the cells were treated with a proteasome inhibitor (carfilzomib), NF-κB inhibitor (BAY11-7082, BAY), AKT inhibitor (LY294002, LY), or β-catenin inhibitor (XAV939), and the cells were treated with six concentrations of 5-FU to calculate the IC50 value from the cell survival curves using the MTT assay. **(B)** NLS-mutated Nrf2 plasmid and/or shZEB1 were transfected into the shNrf2-HCT116 clone. After 24 h, cells were treated with six concentrations of 5-FU to calculate IC50 value by the MTT assay. **(C)** Western blotting analysis was performed to evaluate the expression of PSMD4, ZEB1, E-cadherin, vimentin, fibronectin, YAP1, and HO-1 following these treatments. The cells were treated with 0.1% DMSO or 10μM 5-FU for 24 h. The cells were subjected to annexin V and PI staining and flow cytometry analysis. The percentage of apoptotic cells, including the Annexin V+/ PI- population (early apoptosis) plus Annexin V+/PI– (late apoptosis/secondary necrosis), was determined by a flow cytometry analysis. Data are expressed as means ± s.d. (n = 3). **(D)** The NLS-mutated Nrf2 plasmid was transfected into the shNrf2-HCT116 stable clone. After 24 h, the cells were treated with three concentrations of carfilzomib. Western blotting analysis was performed to evaluate the expression of PSMD4, ZEB1, E-cadherin, vimentin, fibronectin, YAP1, and HO-1 in the stable clone subjected to these treatments. **(E)** The shNrf2-HCT116 stable clone was transfected with shp65, Shβ-catenin, shZEB1, and/or NLS-mutated Nrf2 plasmid. Western blotting analysis was performed to evaluate the expression of PSMD4, ZEB1, E-cadherin, vimentin, fibronectin, YAP1, and HO-1 in this clone subjected to these treatments. The cells were subjected to annexin V and PI staining and flow cytometry analysis. The percentage of apoptotic cells including the Annexin V+/ PI- population (early apoptosis) plus Annexin V+/PI– (late apoptosis/secondary necrosis) was determined by flow cytometry analysis. Data are expressed as means ± s.d. (n = 3).

The cNrf2 promotes tumor invasion via the NF-κB/AKT/β-catenin cascade^7^. We therefore used the stable NLS-mutated Nrf2-transfected shNrf2HCT116 clone to explore which inhibitor of the cascade could have a greater inhibitory effect than carfilzomib on cNrf2-mediated 5-FU resistance (Figure 2A left panel). The IC20 value of each inhibitor was first obtained from MTT assays, and then the IC20 value of each inhibitor was used to examine their IC50 values in the NLS-mutated Nrf2-transfected HCT116 clone (Figure 2A lower right panel). The MTT assays showed that carfilzomib had the lowest IC50 value for 5-FU in the NLS-mutated Nrf2-transfected shNrf2HCT116 clone, followed by an inhibitor for β-catenin transcriptional activity XAV939, an NF-κB inhibitor BAY11-7082 (BAY), and an AKT inhibitor LY-294002 (LY) (Figure 2A upper right panel). The increase in the IC50 value in the NLS-mutated Nrf2-tranfected shNrf2HCT116 clone was completely reversed by ZEB1 silencing (Figure 2B). Western blotting indicated that the expressions of PSMD4, ZEB1, fibronectin, and vimentin were nearly concomitantly reduced by carfilzomib, BAY, LY, and XAV939 treatments, respectively, but E-cadherin expression was markedly elevated by these treatments (Figure 2C upper panel). Annexin-PI staining indicated that the percentage of apoptotic cells induced by 5-FU was significantly elevated by these treatments, when compared with the clone without any treatment (Figure 2C lower panel).

The dose-dependent inhibition of PSMD4, ZEB1, fibronectin, and vimentin expression and the opposite effect on E-cadherin expression by various concentrations of carfilzomib were confirmed in the NLS-mutated Nrf2-transfected shNrf2HCT116 clone (Figure 2D). Similar changes were observed in the NLS-mutated Nrf2-transfected shNrf2HCT116 clone transfected with shPSMD4, shp65, shβ-catenin, or shZEB1 (Figure 2E). These results indicated that activation of the NF-κB/AKT/β-catenin cascade by cNrf2-induced PSMD4 may be responsible for 5-FU resistance, modulated by increased ZEB1 expression.

### Carfilzomib can efficiently suppress the tumor burden induced by injection of the stable NLS-mutated Nrf2-transfected shNrf2HCT116 clone into nude mice

**Figure 3:**
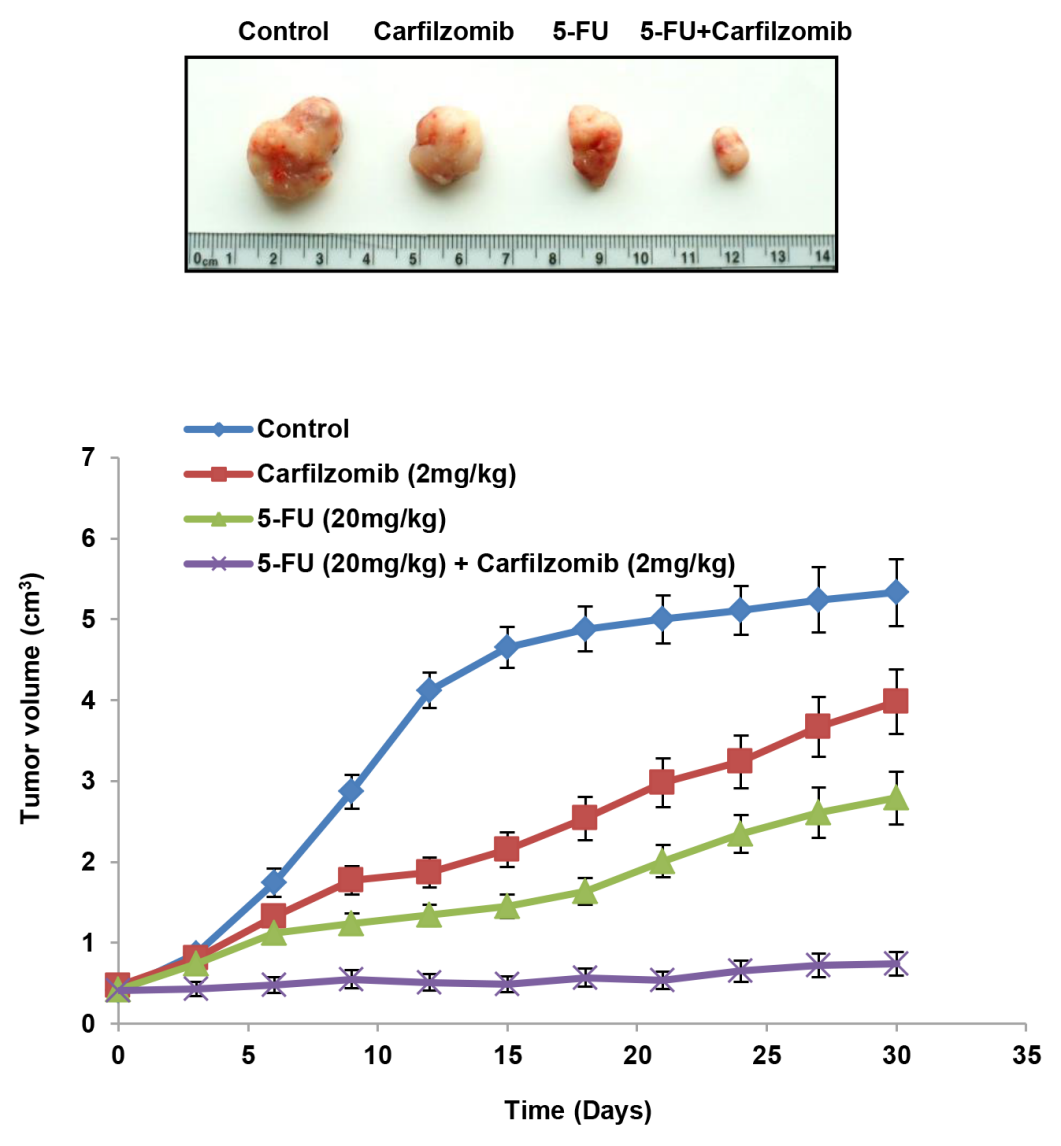
Carfilzomib efficiently suppresses tumor growth induced by the NLS-mutated Nrf2-transfected shNrf2HCT116 clone in nude mice The nude mice were subcutaneously injected with the NLS-mutated Nrf2 shNrf2-HCT116 stable clone (1 × 10^6^ cells). After 7 days, the mice were treated with carfilzomib (2mg/kg) or 5-FU (20mg/kg), singly or in combination, by peritoneal injection. The representative tumor burdens in the four groups are illustrated. The tumor volume in the nude mice of each group was measured at 3-day intervals from day 9 to day 27. Mean ± S.E.M. values (cm^3^) were calculated from the tumor volume of five nude mice in each group.

We performed a preclinical animal model to verify whether carfilzomib could suppress tumor growth induced by the NLS-mutated Nrf2 shNrf2HCT116 clone in nude mice. Mice were randomly divided into four groups of five mice. Each mouse was subcutaneously injected with the NLS-mutated shNrf2HCT116 clone (1 × 10^6^ cells) on day 0 and then given a peritoneal injection of 5-FU, singly or in combination with carfilzomib, on days 7, 14, 21, and 28. All mice were sacrificed on day 30 and their tumors were removed to measure the tumor volumes. The representative tumor burdens for each group of mice are presented in Figure 3 (upper panel). The tumor burden induced by transfection of the shNrf2HCT116 clone with the NLS-mutated Nrf2 was almost completely suppressed by the 5-FU plus carfilzomib combination treatment, but the tumor burden was only partially reduced by treatment with carfilzomib or 5-FU alone when compared with the control group (Figure 3 lower panel). These results strongly support the observations of the cell model and indicate that carfilzomib may overcome 5-FU resistance, thereby improving drug sensitivity and, in turn, efficiently suppressing tumor growth.

### Patients with cNrf2 tumors had a more unfavorable response to 5-FU-based chemotherapy when compared to patients with c/nNrf2 both negative or with c/nNrf2 both positive tumors

We collected tumor samples from 59 colorectal cancer patients who had undergone 5-FU-based chemotherapy to examine the possibility that their tumor cNrf2 expression could be associated with their 5-FU-based chemotherapeutic response. The expression of Nrf2, PSMD4, p-p65, p-AKT, and nuclear β-catenin was evaluated by immunohistochemistry and data obtained from our previous report (Supplementary Figure 2) [7, 19]. In this study population, an association between Nrf2 expression and clinical parameters was only observed for tumor size (T value) (Supplementary Table 2). The cNrf2 (C+/N-) tumors were larger than the c/nNrf2 both negative (C-/N-) or c/nNrf2 both positive (C+/N+) tumors (52.9% vs. 16.7% vs. 29.4%, respectively; P = 0.036). PSMD4 positive tumors were more common in cNrf2-positive tumors than in Nrf2-negative and c/nNrf2 tumors (P = 0.042; Supplementary Table 2). A higher prevalence of an unfavorable response to 5-FU-based chemotherapy was observed in patients with cNrf2 tumors than with c/nNrf2 both negative or with c/nNrf2 both positive tumors (46.2% vs. 83.3% vs. 85.7%, respectively; P = 0.033; Table 1). A higher frequency of an unfavorable response to 5-FU-based chemotherapy was also observed in patients with PSMD4-positive, p-p65-positive, and nuclear β-catenin-positive tumors than with their counterparts (42.9% vs. 13.8%, P = 0.015 for PSMD4; 37.5% vs. 10.5%, P = 0.033 for p-p65; 47.6% vs. 18.4%, P = 0.018 for nuclear β-catenin; Table 1). Conversely, PSMD4-negative tumors were more prevalent in c/nNrf2 tumors than in Nrf2-negative and cNrf2-positive tumors. These observations from colorectal cancer patients provided support for the findings from cell and animal models, suggesting that cNrf2-induced PSMD4 may confer 5-FU resistance in colon cancer cells via the NF-κB/AKT/β-catenin cascade.

**Table 1:** Association of Nrf2 expression with tumor response to 5-FU-based chemotherapy in colorectal cancer patients

		Tumor Response		
	Patient No.	Unfavorable(%)	Favorable (%)	*P*
Nrf2				
Negative	12	2(16.7)	10(83.3)	0.033
cNrf2	26	12(46.2)	14(53.8)	
c/nNrf2	21	3(14.3)	18(85.7)	
PSMD4				
Negative	29	4(13.8)	25(86.5)	0.012
Positive	30	13(43.3)	17(56.7)	
p-p65				
Negative	19	2(10.5)	17(89.5)	0.033
Positive	40	15(37.5)	25(62.5)	
p-Akt				
Negative	31	7(22.6)	24(77.4)	0.266
Positive	28	10(35.7)	18(64.3)	
Nucleus β-catenin				
Negative	38	7(18.4)	31(81.6)	0.018
Positive	21	10(47.6)	11(52.4)	

Fifty-nine out of 160 patients were available for the retrospective study to examine the association with the tumor response to 5-FU-based chemotherapy in colorectal cancer patients.

Nrf2, PSMD4, p-p65, p-Akt, and nucleus nucleus β-catenin expression in colorectal tumors evaluated by immunohistochemistry obtained from our previous reports.

Negative: C-/N-, cNrf2: C+/N-, c/nNrf2: C+/N+

## DISCUSSION

Nrf2 overexpression is known to confer drug resistance in various cancers [11, 12, 15–17], including colorectal cancer [14]. The bulk of the existing literature indicates that activation of the Nrf2/ARE pathway promotes antioxidant and detoxified gene expressions, thereby conferring chemoresistance via decreases in ROS-induced DNA damage and apoptosis [8–11]. For example, oxaliplatin activates the Keap1/Nrf2 pathway and confers protection against the cytotoxicity of anticancer drugs in colon cancer cells [20]. The expression of nNrf2 and cNrf2 observed in the NLS-WT Nrf2-transfected shNrf2HCT116 clone revealed the possibility that Nrf2 overexpression in tumor cells may result in the shuttling of Nrf2 between the nucleus and cytoplasm [7], and that the two localizations of Nrf2 might trigger different mechanisms for modulating cell survival and apoptosis.

Consistent with previous studies [10, 21, 22], the IC50 value was markedly decreased in Nrf2-knockdown cells when compared with the IC50 observed in the parental HCT116 cells (4.4 μM vs. 7.1 μM, respectively; Figure 1A). However, the IC50 value for the NLS-WT Nrf2 overexpressing shNrf2HCT116 cells was similar to that of the parental HCT116 cells (7.8 μM vs. 7.1 μM, respectively; Figure 1A). Interestingly, the highest the IC50 value for 5-FU was observed for the NLS-mutated Nrf2-transfected cells when compared with NLS-WT Nrf2-transfected shNrf2HCT116 clone and the parental HCT116 cells (16.3 μM vs. 7.8 μM vs. 7.1 μM, respectively; Figure 1A). A higher IC50 value was also observed for oxaliplatin in the NLS-mutated Nrf2 transfected cells when compared with the IC50 for 5-FU (33.7 μM vs. 16.3 μM, respectively); however, this difference needs further investigation. Nevertheless, the data indicate that cNrf2 may play a more important role than nNrf2 in resistance to both 5-FU and oxaliplatin in colon cancer cells. Mechanistic studies demonstrated that Nrf2-mediated PSMD4 expression may promote more than just colony formation, cell invasion, and soft agar growth, and may also confer 5-FU resistance in colon cancer cells via the NF-κB/AKT/β-catenin/ZEB1 cascade. The possible mechanistic action of cNrf2-mediated PSMD4 expression in chemoresistance was proposed in Supplementary Figure 3. Our retrospective study of a small subset of colorectal cancer patients (n = 59) supported the possibility that cNrf2 could confer 5-FU resistance by the mechanism suggested by the cell and animal models.

Proteasome inhibitors have known to inhibit NF-κB signaling via suppressing the degradation of its inhibitor IκB. Consequently, the tumor suppressor p53 was stabilized by inactivation of NF-κB signaling and in turn, shift the pro-apoptotic/anti-apoptotic balance in the Bcl-2 family proteins to lead cell death [22]. The new-generation proteasomal inhibitor carfilzomib has known to overcome the resistance of the older-generation proteasomal inhibitor bortezomib in the treatment of multiple myeloma and mantle cell lymphoma [23–25]. A simple practice guide for dose conversion between animals and human was used to calculate the dose of carfilzomib used in our animal model and a phase ib/2 PX-171-007 clinical trial [26]. The dose of carfilzomib used our animal model was slightly higher than patients receiving carfilzomib who had better outcome and maximum tolerated dose (1.62 mg/kg vs. 1.51 mg/kg) [27]. Consistent with the present study, increased proteasomal subunit S5a (PSMD4) protein expression and proteasomal activity in colon cancer were related to an enhanced activation of Nrf2 [28]. The Nrf2-dependent PSMD4 expression conferred protection from the apoptosis triggered by tumor necrosis factor-related apoptosis-inducing ligands [28]. Inhibition of the Nrf2 transcription factor by the alkaloid trigonelline renders pancreatic cancer cells more susceptible to apoptosis through decreases in PSMD4 expression and proteasomal activity [29]. Nrf2-mediated PSMD4 expression occurs predominately through increased cytoplasmic localization of Nrf2 by CRM1 elevation due to PSMD4-mediated degradation of p53 [7].

In the present study, the induction of PSMD4 by cNrf2 may have conferred 5-FU resistance by decreasing E-cadherin expression via the NF-κB/AKT/β-catenin/ZEB1 cascade. Therefore, the chemoresistance mediated by cNrf2-induced PSMD4 expression in colorectal cancer may occur predominately through the epithelial-to-mesenchymal transition (EMT). This would be consistent with a large body of literature that implicates NF-κB, β-catenin, and ZEB1 in EMT-mediated chemoresistance [12, 30–36]. Unfortunately, no inhibitor for these three molecules is yet available for clinical use. However, carfilzomib enhances doxorubicin-induced cytotoxicity and apoptosis in breast cancer [37], and when combined with tyrosine kinase inhibitors, carfilzomib may enhance tyrosine kinase inhibitor sensitivities in chronic myeloid leukemia [38]. Our findings presented here provide evidence that carfilzomib may overcome the 5-FU resistance in colorectal cancer induced by the NF-κB/β-catenin/ZEB1 activation that is mediated by cNrf2-induced PSMD4 expression.

In summary, cNrf2 may play a more important role than nNrf2 in the chemoresistance of colorectal cancer. PSMD4 may represent a useful molecular target for overcoming cNrf2-mediated 5-FU resistance in colorectal cancer. Therefore, we suggest that cNrf2 may have the potential to act as a reliable biomarker of poor outcome and chemoresistance in colorectal cancer patients. Moreover, the cell and animal models presented here strongly support a clinical use of carfilzomib for improving tumor regression and the chemotherapeutic response in colorectal cancer patients who harbor cNrf2 expressing tumors.

## MATERIALS AND METHODS

### Study subjects

A total of 59 patients with primary colorectal cancer who underwent 5-FU-based chemotherapy were enrolled in this retrospective study. All patients were unrelated ethnic Chinese persons and residents of central Taiwan. From 2000 to 2007, all patients with primary colorectal cancer were recruited from the Colorectal Division, Department of Surgery, Chung Shan Medical University Hospital, after providing written informed consent approved by the Institutional Review Board (TMU201501036). No patients had received chemotherapy or radiotherapy before surgical resection. The overall survival of patients was based on the date of surgery. A series of examinations for pathological stages was conducted for each case by board certified pathologists. Information pertaining to personal characteristics was collected from hospital reports.

### Cell lines

The CCM2, CCM3, LoVo, and HT29 cells were kindly provided by Drs. W.S. Chang and S.G. Shiah (National Institute of Cancer Research, National Helath Research Institutes, Miaoli, Taiwan). The HCT116 and HCT116 p53^-/-^ cell lines were kindly provided by Dr. C.C. Chang (Institute of Biomedical Sciences, National Chung Hsing University, Taichung, Taiwan). The CCM2, CCM3, HC15, HCT116, and HCT116 p53-/- cells were maintained in RPMI-1640 (HyClone Logan, UT, USA). The LoVo cells were maintained in F12-K (HyClone Logan, UT, USA). The medium contained 10% FBS supplemented with penicillin (100 U/ml) and streptomycin (100 mg/ml). Cells were grown at 37°C in a humidified incubator at 5% CO_2_. Cells were cultured and stored according to the suppliers’ instructions and used at passages 5 to 20. Once resuscitated, cell lines were routinely authenticated (once every 6 months, cells were last tested in December 2015) through cell morphology monitoring, growth curve analysis, species verification by isoenzymology and karyotyping, identity verification using short tandem repeat profiling analysis, and contamination checks

### Antibodies

Antibodies to Nrf2, p-AKT (S473), and HO-1 (GTX61763, GTX50128, and GTX101147, respectively) were purchased from Genetex (Irvine, CA, USA). All other antibodies were purchased from Santa Cruz Biotechnology (Dallas, TX, USA).

### Immunohistochemistry analysis

Expression of Nrf2, PSMD4, p-p65, p-Akt, and nuclear β-catenin in patients’ colorectal tumors was evaluated by immunohistochemistry using specific antibodies. The immunostaining results were obtained from our previous report [7, 19]. Specimens were formalin fixed and paraffin embedded. In brief, 3 μm sections were cut, mounted on glass, and dried overnight at 37 °C. All sections were then deparaffinized in xylene, rehydrated through a graded alcohol series, and washed in phosphate-buffered saline. This buffer was used for all subsequent washes. Sections were heated in a microwave oven twice for 5 min in citrate buffer (pH 6.0). The sections were then incubated with the appropriate antibody for 60 min at room temperature, followed by conventional streptavidin peroxidase detection (LSAB Kit K675, DAKO, Carpinteria, CA, USA). Signals were developed with 3, 3’-diaminobenzidine for 5 min and counterstained with hematoxylin. Negative controls were obtained by leaving out the primary antibody. The intensities of the signals were evaluated independently by three observers.

### Plasmid construction

Nrf2 cDNA was cloned into pcDNA3.1 Zeo(+) (Invitrogen, Carlsbad, CA, USA) by PCR amplification with newly created XhoI and BamHI sites attached onto the 5’ends of the forward and reverse Nrf2 primers, using H116 cDNA as a template. The NLS-mutated Nrf2 was generated using the QuickChange site-directed mutagenesis system (Stratagene, San Diego, CA, USA). Mutant NLS primers are presented in Supplementary Table 1 and are based on a previous report [18]. The shRNA was purchased from the National RNAi Core Facility, Academia Sinica, Taiwan, and the shRNA target sequences are presented in Supplementary Table 1.

### Plasmid transfection reaction

Different concentrations of expression plasmids were transiently transfected into colon cancer cells (1×10^6^) using the Turbofect transfection reagents (Thermo, Waltham, MA, USA). After 48 h, the cells were harvested and whole-cell extracts were assayed in subsequent experiments.

### Selection of HCT116 stable clones

The Nrf2 shRNA plasmids (10 μg) were mixed with Turbofect transfection reagents (Thermo, Waltham, MA, USA) and added to 1×10^5^ HCT116 cells. After 48 h, stable transfectants for Nrf2 shRNA were selected using 1μg/ml puromycin (Sigma-Aldrich, St. Louis, MO, USA). The selection medium was replaced every 3 days for 3 weeks. Interference with Nrf2 expression was confirmed by western blotting.

### The 3-(4,5-cimethylthiazol-2-yl)-2,5-diphenyl tetrazolium bromide (MTT) cytotoxicity assay

The cell lines were cultured in 96-well flat-bottomed microtiter plates supplemented with RPMI 1640 and DMEM containing 10% heat-inactivated fetal bovine serum, 100 units/mL penicillin, and 100 units/mL streptomycin. The cells were incubated in a humidified atmosphere containing 95% air and 5% CO_2_ at 37°C until they reached the exponential growth phase. The cells pretreated with an miR mimic, inhibitor, shRNAs, or p53 and Bcl-2 overexpression plasmids for 24 h, followed by cisplatin treatment (0, 2, 4, 8, 16, 32 μM). After 48 h incubation, the *in vitro* cytotoxic effects of these treatments were determined by MTT assays (at 570 nm).

### Annexin-V/PI staining

The cells were collected by trypsinization and centrifugation at 1,000g for 5 minutes. Following resuspension in binding buffer (10 mmol/L HEPES-NaOH, 140 mmol/L NaCl, 2.5 mmol/L CaCl_2_) at a final cell density of 1 to 2 ×10^6^ cells/mL, 100 μL of a single-cell suspension (1-2 × 10^5^ cells) was incubated with 5 μL Annexin-V–FITC and 5 μL propidium iodide (PI) for 15 minutes at room temperature in the dark. After addition of 400 Ml of binding buffer, the samples were analyzed with a BD FACS Calibur flow cytometer (BD Biosciences) within 1 hour. For each sample, 10,000 events were counted.

### *In vivo* animal therapeutic analysis

All animal studies were approved by the Institutional Animal Care and Use Committee at Taipei Medical University. These animals were maintained in individual ventilated cages according to the guidelines established in “Guide For The Care and Use of Laboratory Animals” prepared by the Committee on Care and Use of Laboratory Animals of the Institute of Laboratory Animal Resources Commission on Life Sciences, National Research Council, U.S.A. (1985). Use of animals has been approved by the Institutional Animal Care and Use Committee of Taipei Medical University, Taipei, Taiwan (LAC-2014-0257). The models of colorectal adenocarcinoma were 4-week-old female BALB/c nude mice (n = 20; supplied by the National Laboratory Animal Center, Taiwan) that were acclimated for 1 week while caged in groups of 5. The mice were housed in SPF conditions and fed a diet of animal chow and water throughout the experiment. Therapeutic experiments on tumor growth were initiated by injecting NLS-mutated Nrf2 HCT116 cells (10^6^ cells in 0.1 mL of PBS) subcutaneously into the backs of 5-week-old female BALB/c nude mice. The xenograft size was measured every three days and the tumor volume was determined as (length × width^2^)/2. When tumors had grown to 0.5 cm^3^, mice were randomized to the following groups: control (DMSO), carfilzomib (2mg/kg), 5-FU (20mg/kg), and a combination of both drugs. Drugs were administered by intraperitoneal injection every 7 days.

### Statistical analysis

Statistical analysis was conducted using the SPSS statistical software program (Version 15.0; SPSS Inc.). Survival plots were generated using the Kaplan–Meier method, and differences between patient groups were determined by the log-rank test. Multivariate Cox regression analysis was conducted to determine survival rate. The analysis was stratified for all known variables (age, gender, and tumor stage) and for protein expression.

## SUPPLEMENTARY MATERIALS FIGURES AND TABLES


