# Supplementary material for: PSMD4 is a novel therapeutic target in chemoresistant colorectal cancer activated by cytoplasmic localization of Nrf2
Source: Oncotarget. 2018 May 29;9(41):26342–52. doi: 10.18632/oncotarget.25254 (PMC5995171; doi:10.18632/oncotarget.25254)
Supplement: Supplementary file 1 [file oncotarget-09-26342-s001.pdf]

PSMD4 is a novel therapeutic target in chemoresistant colorectal cancer activated by cytoplasmic localization of Nrf2

SUPPLEMENTARY MATERIALS

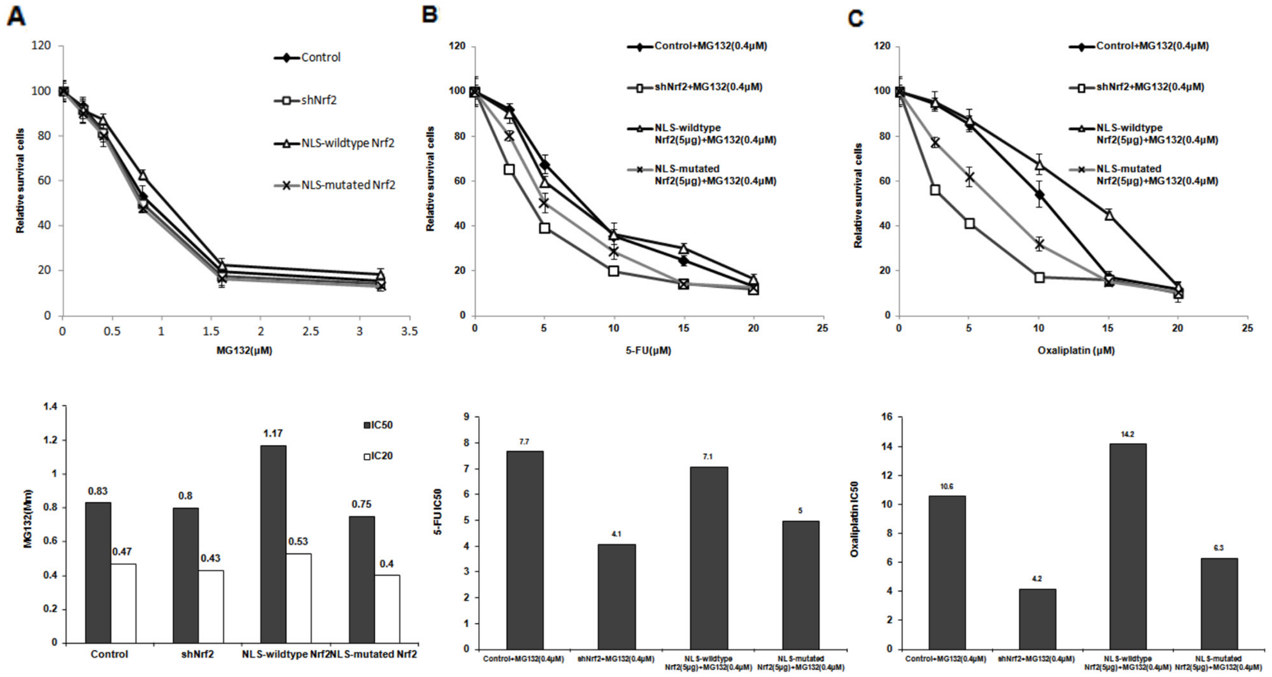

Supplementary Figure 1: **(A)** NLS-WT Nrf2 or a NLS-mutated Nrf2 expression vector was transfected into a shNrf2-HCT116 stable clone. Cells were treated with six concentrations of MG132 to calculate the IC50 and IC20 value from the dose-response survival curve determined by the MTT assay. NLS-WT Nrf2 and NLS-mutated Nrf2 plasmids were transfected into the stable shNrf2-HCT116 clone. After 24 h, cells were treated with MG132 (0.4 μM) and then with six concentrations of **(B)** 5-FU and **(C)** oxaliplatinto calculate the IC50 value from the cell survival curves using the MTT assay.

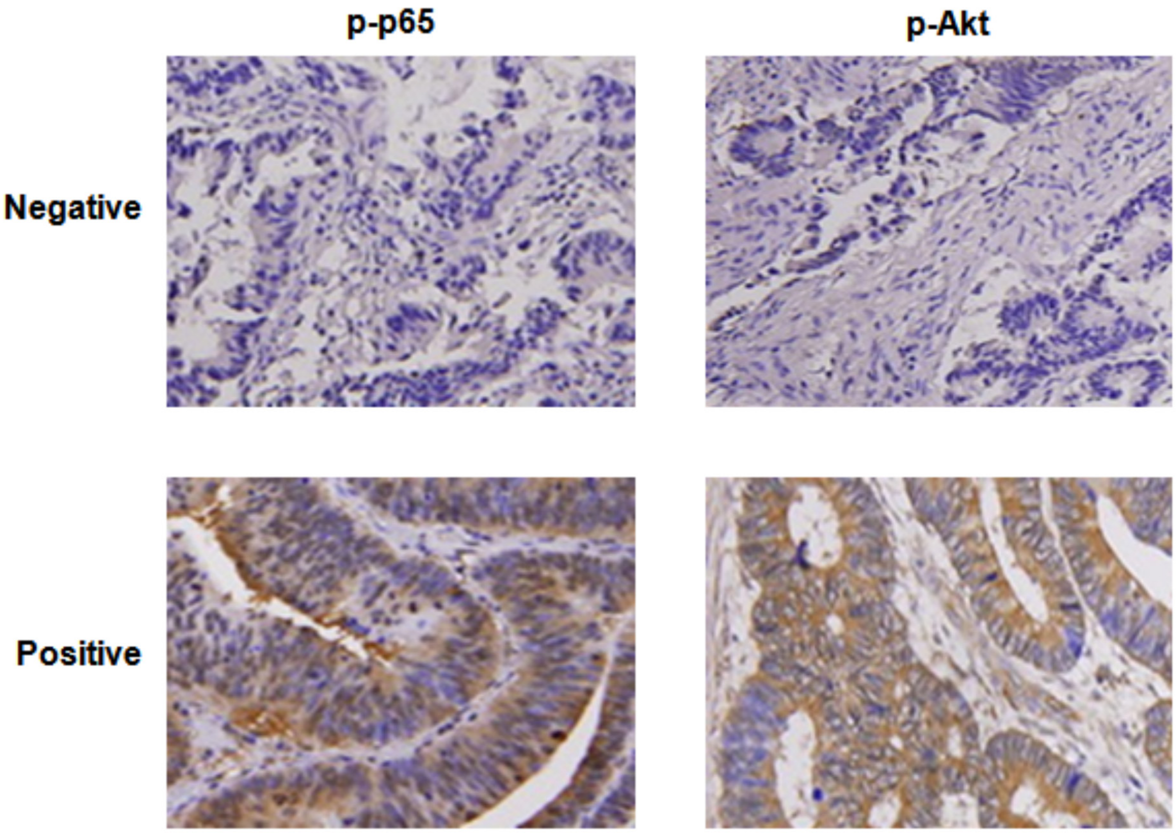

Supplementary Figure 2: Representative immunostaining results of p-p65 and p-Akt expression in tumors from colorectal cancer patients.

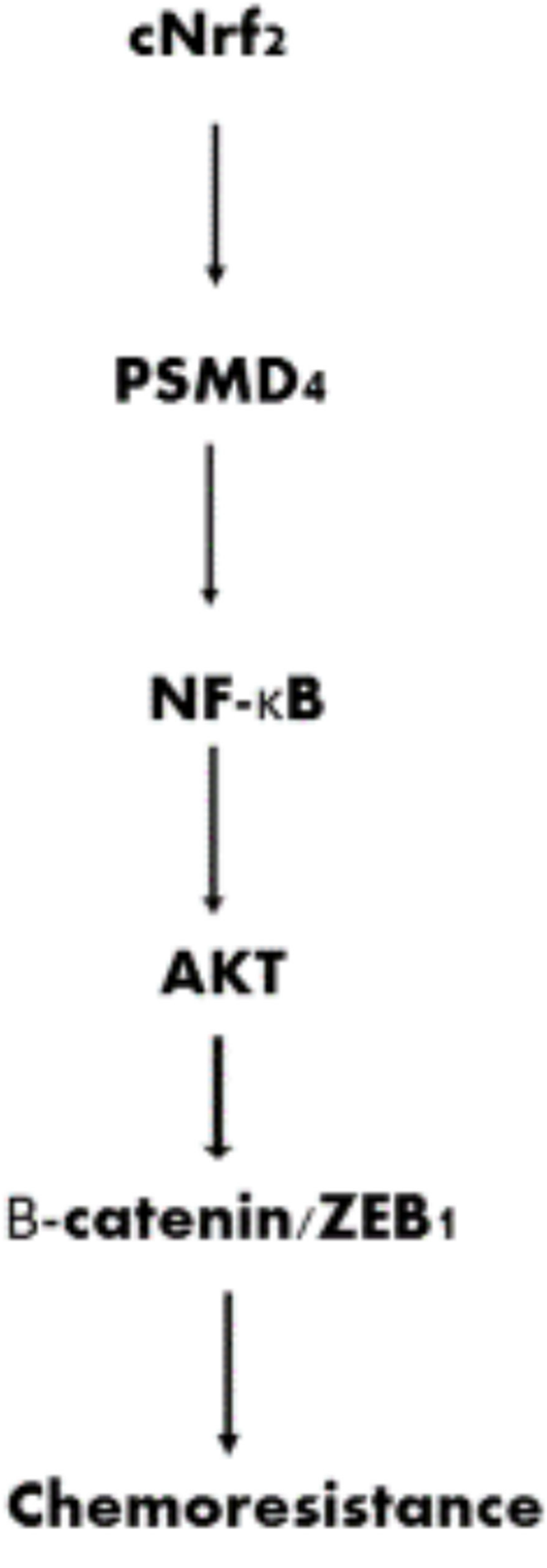

Supplementary Figure 3: The possible mechanistic action of cNrf2 on chemoresistance in colorectal cancer.

Supplementary Table 1: The primers and shRNA target sequences used in the present study

Target gene	Sequence
**Construct**	
Nrf2 Forward	5’-CTCGAGATGATGGACTTGGAGCTGCCGCC-3’
Nrf2 Reverse	5’-GGATCCCTAGTTTTTCTTAACATCTGGCT-3’
**Site-directed mutagenesis**	
Mutant NLS Forward	5’-agcgacagaaggactatgagctggaaGCacagGCCGCactcgaaaaggaaagacaagagcaactc- 3’
Mutant NLS Reverse	5’-gagttgctcttgtctttccttttcgagtGCGGCctgtGCttccagctcatagtccttctgtcgct-3’
**RNAi target**	
shPSMD4	5’-ACAATGAAGCCATTCGAAATG-3’
shZEB1	5’- CCTCTCTGAAAGAACACATTA-3’
shβ-catenin	5’-CCTTTAGCTGTATTGTCTGAA-3’

Supplementary Table 2: Relationships of Nrf2 expression with clinico-pathological parameters in colorectal cancer patients

		Nrf2			
**Characteristics**	**Patient No.**	**Negative(%)**	**cNrf2 (%)**	**c/nNrf2 (%)**	***P* value**
**Total patients**	59	12 (20.3)	26 (44.1)	21 (35.6)	
**Age**					
≤64	29	4 (13.8)	13 (44.8)	12 (41.4)	0.418
>64	30	8 (26.7)	13 (43.3)	9 (30.0)	
**Gender**					
Female	24	6 (25.0)	11 (45.8)	7 (29.2)	0.628
Male	35	6 (17.1)	15 (42.9)	14 (40.0)	
**Smoking status**					
Nonsmoking	38	6 (15.8)	19 (50.0)	13 (34.2)	0.369
Smoking	21	6 (28.6)	7 (33.3)	8 (38.1)	
**Stage**					
I+II	25	6 (24.0)	8 (32.0)	11 (44.0)	0.275
III+IV	34	6 (17.6)	18 (52.9)	10 (29.4)	
**T**					
1+2	5	3 (60.0)	0 (0.0)	2 (40.0)	0.036
3+4	54	9 (16.7)	26 (48.1)	19 (35.2)	
**N**					
0	29	7 (24.1)	10 (34.5)	12 (41.4)	0.345
1+2	30	5 (16.7)	16 (53.3)	9 (30.0)	
**M**					
No	51	12 (23.5)	20 (39.2)	19 (37.3)	0.123
Yes	8	0 (0.0)	6 (75.0)	2 (25.0)	
**PSMD4**					
Negative	29	8 (27.6)	8 (27.6)	13 (44.8)	0.042
Positive	30	4 (13.3)	18 (60.0)	8 (26.7)	

Negative: C-/N-
cNrf2: C+/N-
c/nNrf2: C+/N+
